# Cysteine Promoted C-Terminal Hydrazinolysis of Native Peptides and Proteins**

**DOI:** 10.1002/anie.201304997

**Published:** 2013-10-09

**Authors:** Anna L Adams, Ben Cowper, Rachel E Morgan, Bhavesh Premdjee, Stephen Caddick, Derek Macmillan

**Affiliations:** Department of Chemistry, University College London20 Gordon Street, London, WC1H 0AJ (United Kingdom)

**Keywords:** acyl transfer, erythropoietin, hydrazides, native chemical ligation, peptides

In 1998, the Ramage group demonstrated that a C-terminal hydrazide of a synthetic peptide could, following diazotization to the corresponding acyl azide, be transformed into usefully functionalized peptides, including thioesters.^1^ A drawback was that certain amino acid residues needed to be protected, but Liu and co-workers more recently showed that peptide and protein hydrazides could be converted into thioesters for use in native chemical ligation (NCL) under optimized conditions without protecting groups.^2^ Protein C-terminal hydrazides are useful products in their own right and allow selective modification of the protein through the uniquely reactive C-terminus.^3^ Until recently,^4^ a protein C-terminal hydrazide had only been obtained by hydrazinolysis of intein fusion precursors.^2^, ^3^ Consequently, it is desirable that complementary routes become available, particularly for proteins that are not anticipated to express as soluble and folded intein fusion proteins.^5^

Previously, we demonstrated that native peptides and proteins could undergo thioester formation across Xaa–Cys motifs by N→S acyl transfer in the absence of inteins.^6^ We reasoned that, as an efficient nucleophile, hydrazine could be a suitable additive for this process, which leads to C-terminal hydrazides by hydrazinolysis of a transient thioester. This may provide a more robust, albeit less direct, route to protein thioesters, as the acyl hydrazide would not hydrolyze under the reaction conditions. Furthermore, conversion of the hydrazide into the thioester appears to be essentially free from hydrolysis when the thioester undergoes NCL in situ.^2^ We first examined a model peptide,^6^ and employed hydrazinium acetate as the hydrazine source in sodium phosphate buffer (0.1 m) at a pH of 5.8 (final pH of ca. 7),^7^ in the presence and absence of MESNa (Table 1).

**Table 1:** Hydrazinolysis of a His–Cys terminated model peptide
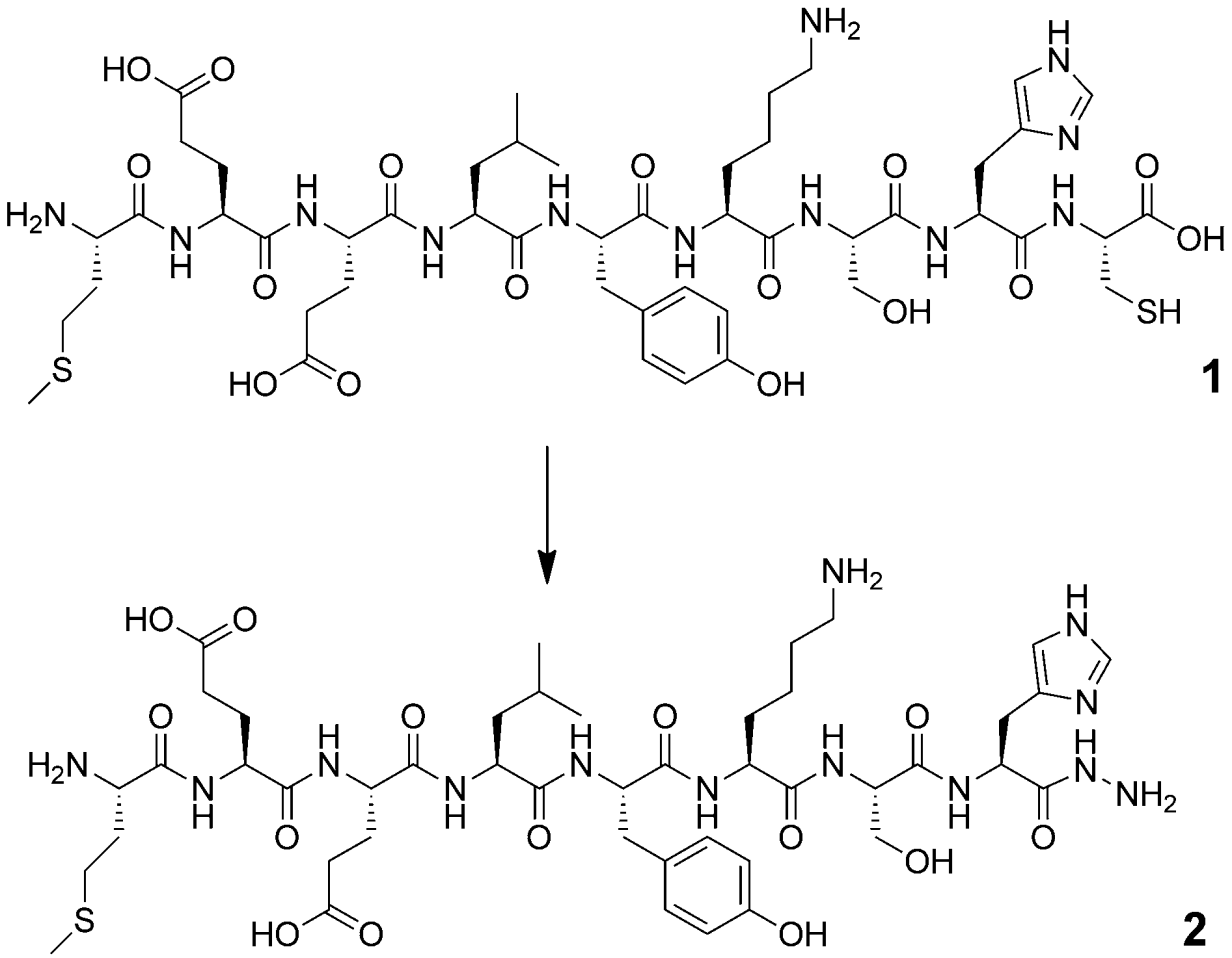

Entry	N_2_H_4_⋅HOAc	*T* [°C]	MESNa [% w/v]	1^[a]^ [%]	
	[% w/v]			24 h	48 h
1	5	60	10	5	0
2	5	50	10	27	5
3	5	40	10	28	19
4	0	50	10	33	30
5	5	50	5	33	21
6	2.5	50	10	–^[b]^	5
7	5	50	0	–^[b]^	30

[a] Because of large differences in the chromophoric properties of **1** and **2**, only consumption of **1** was quantified by HPLC. [b] Not determined.

HPLC and LC-MS analysis of the reaction mixture indicated that hydrazinolysis of a His–Cys motif proceeded efficiently when using N_2_H_4_⋅HOAc (5 % w/v). The reaction was nearly complete within 24 hours at 60 °C (Table 1, entry 1), which confirms that hydrazine was highly effective at intercepting thioester intermediates, allowing us to investigate lower reaction temperatures (entries 2 and 3). The reaction proceeded very smoothly, and only **1** and **2** were observed, except for: 1) when the reaction was conducted in the absence of hydrazinium acetate (entry 4), where the MESNa thioester was the major product, and 2) in the absence of MESNa (entry 7), where significant deterioration in the quality of the sample was observed over time by LC-MS analysis. Interestingly, although thioester intermediates were not observed in the presence of hydrazinium acetate (5 % w/v) by either LC-MS or ^13^C NMR analysis, when using a ^13^C labeled Gly–Cys terminated precursor (Supporting Information, Figure S1), MESNa appeared to be required. In the absence of MESNa, dimerization of **1** and methionine oxidation were the most prevalent reactions. In contrast to thioester formation, a prolonged reaction time did not exacerbate product hydrolysis. Hydrazide **2** was isolated and smoothly converted into a C-terminal MESNa thioester within one hour at −10 °C by using the optimized conditions described by Liu and co-workers (Figure S2).^2^

In a further model study, the 29-residue glycopeptide **3** (Figure 1), which corresponds to residues 1–29 of erythropoietin (EPO), was exposed to hydrazinolysis and thioester formation in a similar fashion. After 48 hours, LC-MS analysis indicated that the hydrazide was formed as the major product, with no evidence that the appended carbohydrate was affected. Furthermore, no hydrazinolysis had occurred across the internal unprotected Ile–Cys motif. This hydrazide was also converted into the corresponding MESNa thioester **4**.

**Figure 1:**
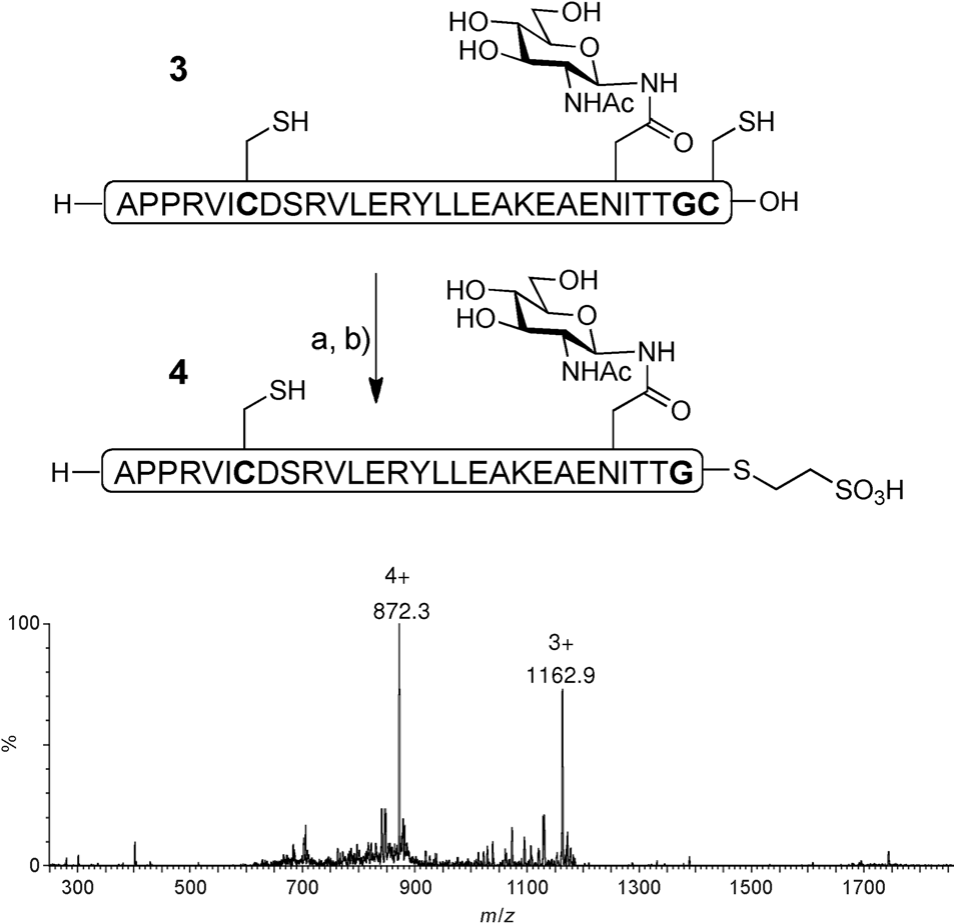
Glycopeptide thioester formation. a) Guanidine⋅HCl (6 m), sodium phosphate (0.1 m), pH 5.8, MESNa (10 % w/v), N_2_H_4_⋅HOAc (5 %), 50 °C, 48 h. b) Guanidine⋅HCl (6 m), sodium phosphate (0.2 m), pH 4, NaNO_2_, −10 °C, 20 min; then MESNa (0.1 m), 1 h. Calcd. *M*=3484.7 Da; obs. *M*=3485.5 Da. Ac=acetyl, MESNa=sodium 2-mercaptoethanesulfonate.

Hydrazinolysis was next applied to the N-terminal “half” of human hepcidin that terminates in a Gly–Cys motif (**5**; Figure 2). Because of the abundance of potentially labile sites, the internal cysteine residues were protected. Peptide **5** underwent efficient hydrazinolysis to afford **6**, which was isolated in 53 % yield. Subsequent one-pot diazotisation/NCL^2^ with peptide **7** gave rise to full-length hepcidin precursor **8** in acceptable yield. Acm deprotection and oxidative folding^8^ then gave rise to hepcidin (**9**). Notably, the potentially sensitive N-terminal aspartic acid residue^9^ was stable under the reaction conditions.

**Figure 2:**
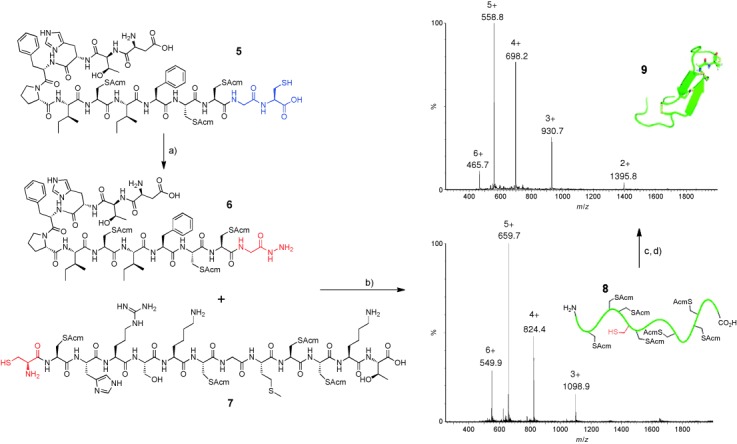
Synthesis of hepcidin from the Gly–Cys terminated precursor **5**. Reagents and conditions: a) Sodium phosphate (0.1 m), pH 5.8, MESNa (10 % w/v), N_2_H_4_⋅HOAc (5 % w/v), guanidine⋅HCl (6 m), 60 °C, 24 h, 53 % yield of isolated product. b) Sodium phosphate (0.2 m), pH 4, NaNO_2_, −10 °C, 20 min; then MPAA (0.1 m), guanidine⋅HCl (6 m), pH 7, 2 h, 44 % yield of isolated product. For **8**: calcd. *M*=3295.0 Da; obs. *M*=3293.8 Da. c) AgOAc, TFA (0.1 %) in H_2_O/MeCN (1:1), DTT, 24 h. d) oxidation.^8^ For **9**: calcd. *M*=2789.4 Da; obs. *M*=2789.4 Da. Acm=acetamidomethyl, DTT=1,4-dithiothreitol, MeCN=acetonitrile, MPAA=4-mercaptophenylacetic acid, TFA=trifluoroacetic acid.

Interestingly, peptides **3** and **5** had previously performed rather poorly in direct thioester formation, and this new method presented a significant improvement. However, these species are available through several alternative means, and a significant advance would entail applications to proteins of biological origin.

Next, human ubiquitin (G76 C) was overexpressed and purified from *E. coli*. Ubiquitin is particularly suitable for model studies, because it contains no native cysteine residues.^10^ The expressed protein was adorned with a TEV-protease-cleavable N-terminal affinity purification tag that was retained throughout the experiments (Figure 3 a). Exposure of this protein to typical reaction conditions for thioester formation resulted in significant product formation within 24 hours at 50 °C (Figure 3 b). As might be expected, a small degree of thioester hydrolysis was apparent after 24 hours. In contrast, exposure to hydrazinium acetate (5 % w/v) facilitated complete conversion into the corresponding hydrazide without hydrolysis after 48 hours at 45 °C (Figure 3 c). For conversion into the thioester, slightly modified reaction conditions to those described by Liu and co-workers were employed. To minimize the amount of denaturation of the expressed protein, MESNa thioester formation was conducted at approximately 0 °C in sodium phosphate buffer rather than at −10 °C in guanidine⋅HCl (6 m; Figure 3 d). Similarly, an MPAA thioester that was formed in situ, as described for peptide **6** (Figure 2), underwent concomitant NCL with **10** to afford C-terminally biotinylated ubiquitin (Figure 3 e). Alternatively, NCL with cysteamine (**11**) provided access to C-terminal aminoethanethiols (Figure 3 e), which have proven useful in recent applications of disulfide directed, site-specific ubiquitination of proteins.^11^ This is the first time that recombinant proteins, such as ubiquitin, have been C-terminally functionalized in this way by purely chemical means, and studies of protein assemblies that bear native ubiquitin isopeptide linkages should be possible with this approach, when it is used in concert with an acyl-transfer auxiliary.^12^ As all of the required reagents for hydrazide and thioester formation are salts, protein-handling steps were essentially performed in a one-pot fashion with the aid of a centrifugal filter (with a cut-off molecular weight of 3 kDa) to exchange buffers between reactions.

**Figure 3:**
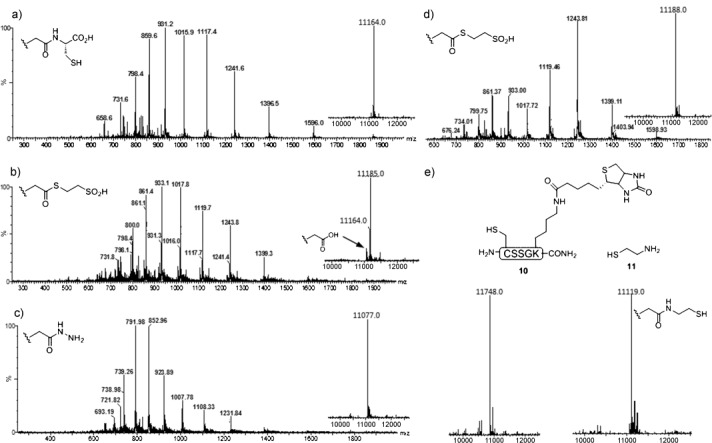
Reactions at the C terminus of G76 C ubiquitin. a) Purified His_6_–ubiquitin; calcd. *M*=1163.0 Da; obs. *M*=11164.0 Da. b) Thioester formation: NaCl (0.1 m), sodium phosphate (0.1 m), pH 5.8, MESNa (10 % w/v), 50 °C, 24 h; calcd. *M*=11184.0 Da; obs. *M*=11185.0 Da. c) Hydrazide formation: NaCl (0.1 m), sodium phosphate (0.1 m), pH 5.8, MESNa (10 % w/v), N_2_H_4_⋅HOAc (5 % w/v), 45 °C, 48 h; calcd. *M*=11074.0 Da; obs. *M*=11077.0 Da. d) Conversion of the hydrazide into the thioester*:* sodium phosphate (0.2 m), pH 4, NaNO_2_, 0 °C, 20 min; then MESNa (0.2 m), pH 7. e) Conversion into the biotinylated analogue and mercaptoethylamide*:* sodium phosphate (0.2 m), pH 4, NaNO_2_, 0 °C, 20 min; then MPAA (0.1 m) containing excess biotinylated peptide or cysteamine, pH 7, 1 h. For C-terminal-biotinylated ubiquitin: calcd. *M*=11748.8 Da; obs. *M*=11748.0 Da. For C-terminal aminoethanethiols: calcd. *M*=11119.0 Da; obs. *M*=11119.0 Da.

We finally examined hydrazinolysis of unglycosylated EPO, a 166-amino acid residue hormone.^13^ Unglycosylated EPO is notoriously insoluble^14^ and also harbors four cysteine residues, with at least two sites in the protein (Gly^28^-Cys^29^ and His^32^-Cys^33^) susceptible to N→S acyl transfer.^4^ ^13^C NMR analysis of labeled thioester precursors confirmed that the rate of hydrazinolysis of terminal Cys carboxylates was, in general, significantly faster than for Cys-carboxamide-terminated samples (Figure S1), and it was hoped that the greater reactivity of terminal Cys carboxylates over internal Cys carboxamides would confer sufficient selectivity on the reaction.

The EPO residues 1–161 were overexpressed and purified from *E. coli* (Figure 4 a). Hydrazinolysis across an A160 GC motif would facilitate C-terminal labeling using short synthetic peptides that comprise residues 161–166. This allows the protein to be modified at the C-terminus through NCL, without the introduction of an additional free Cys residue, by using the native cysteine (Cys 161) as the ligation site. The poorly soluble EPO fragment was solubilized in guanidine⋅HCl (6 m) under reducing conditions, and hydrazinolysis, while slow, proceeded smoothly when performed at 45 °C. Notably, and in contrast to ubiquitin, hydrazinolysis appeared to cease at approximately 40 % conversion after 48 hours, which is not an uncommon observation for samples that require the presence of guanidine⋅HCl (6 m) for sufficient solubility. Furthermore, continuing the reaction beyond this time resulted in deterioration of the sample quality. However, exchanging the low-molecular-weight reagents after 48 hours by using a centrifugal filter allowed the reaction to be run to near completion, without significant sample deterioration. In agreement with the results in Table 1 (entry 7), the presence of reduced MESNa is important for maintaining a homogeneous protein sample. Fragmentation of the protein across the internal His–Cys and Gly–Cys sequences was observed by LC-MS, although not to such an extent that it precluded product formation. SDS-PAGE analysis of the reaction mixture after 96 hours indicated that the majority of the protein had remained intact (Figure 4 b). It is likely that the efficiency of this reaction could be further improved by simple mutation of the Gly and His residues of these Xaa–Cys motifs, without loss of bioactivity.^15^

**Figure 4:**
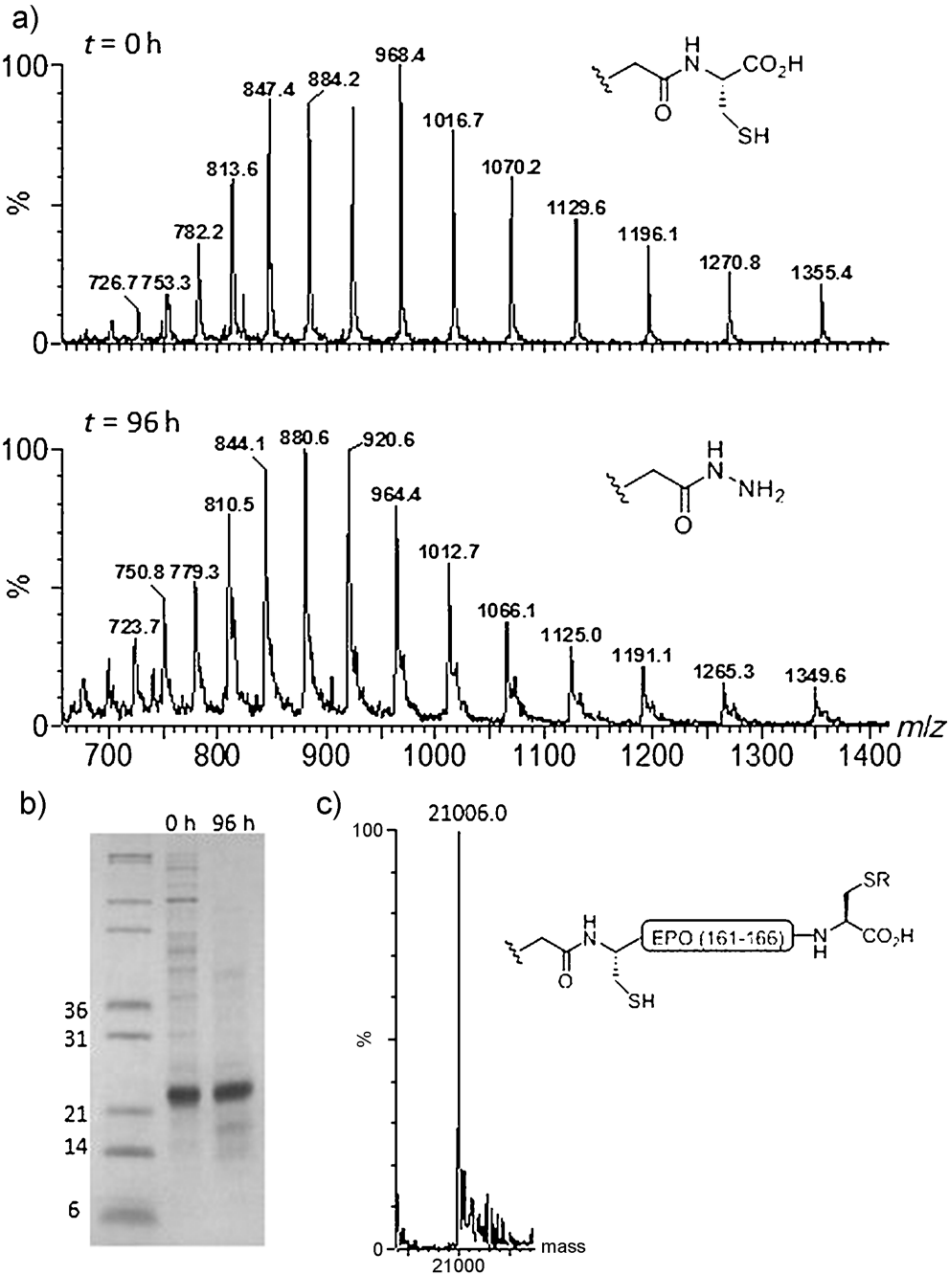
a) MS analysis of the EPO hydrazinolysis reaction at *t*=0 h and *t*=96 h. b) SDS-PAGE analysis of the reaction mixture at *t*=0 h and *t*=96 h. c) Mass spectrum of the product after thioester formation/NCL with the peptide H-CRTGDRC-OH (in this model reaction, R=H). Calcd. *M*=21006.0 Da; obs. *M*=21006.0 Da. SDS-PAGE=sodium dodecylsulfate-polyacrylamide gel electrophoresis.

In a model ligation reaction, successful thioester formation and in situ NCL with Cys terminated EPO residues 161–166 (Figure 4 c) was observed, thus opening the door to selective C-terminal modification of bacterially produced EPO, which has not been previously achieved. It is envisaged that prior alkylation of the C-terminal Cys residue of the short peptide will give rise to a variety of C-terminal-labeled EPO analogues.

In conclusion, hydrazinolysis across His–Cys and Gly–Cys motifs that are installed at the C terminus of native peptides and proteins was demonstrated for the first time, and culminated in a method for hydrazinolysis/thioesterification/NCL. For model peptides, hydrazinolysis occurred more rapidly than thioester formation, and no hydrolysis was observed. On expanding the application of this method to recombinant proteins, even challenging samples with poor solubility could be processed to C-terminal hydrazides, and then thioesters. This new development offers a valuable complementary method for the production of C-terminal-labeled proteins, for which the synthetic options are still limited.

## Supplementary material

As a service to our authors and readers, this journal provides supporting information supplied by the authors. Such materials are peer reviewed and may be re-organized for online delivery, but are not copy-edited or typeset. Technical support issues arising from supporting information (other than missing files) should be addressed to the authors.
