# Supplementary material for: Cysteine Promoted C-Terminal Hydrazinolysis of Native Peptides and Proteins
Source: Angew Chem Int Ed Engl. 2013 Oct 9;52(49):13062–6. doi: 10.1002/anie.201304997 (PMC4065347; doi:10.1002/anie.201304997)
Supplement: Supplementary file 1 [file anie0052-13062-SD1.pdf]

Angewandte Chemie
Eine Zeitschrift der Gesellschaft Deutscher Chemiker

Supporting Information

© Wiley-VCH 2013

69451 Weinheim, Germany

Cysteine Promoted C-Terminal Hydrazinolysis of Native Peptides and Proteins**

*Anna L. Adams, Ben Cowper, Rachel E. Morgan, Bhavesh Premdjee, Stephen Caddick, and Derek Macmillan**

anie_201304997_sm_miscellaneous_information.pdf

Materials and methods

Peptide synthesis was carried out using Rink amide MBHA resin for the production of peptide Cys carboxamides (loading = 0.64 mmol/g) and NovaSyn TGT resin pre-loaded with Fmoc-Cys(Trt)-OH for synthesis of C-terminal Cys carboxylic acids (loading = 0.21 mmol/g). NovaSyn TGT resin pre-loaded with Fmoc-Thr(tBu)-OH (loading = 0.20 mmol/g) was employed for the synthesis of hepcidin residues 13-25. All resins and Fmoc amino acids were purchased from Merck Biosciences. Mass spectra were obtained on Waters uPLC/SQD-LC series electrospray mass spectrometer. LC-MS was performed using a gradient of 5-95% acetonitrile containing 0.1% formic acid over 10 minutes (flow rate of 0.6 mL/min). Semi-preparative HPLC was performed using a Phenomenex LUNA C18 column and a gradient of 10-60% acetonitrile containing 0.1% TFA over 50 minutes (flow rate of 4.0 mL/min). All other chemical reagents were obtained form Sigma.

General peptide synthesis procedure: For model experiments peptide C-terminal carboxylic acids were generally prepared on a 0.05 mmol scale on pre-loaded Fmoc-Cys(Trt)-NovaSyn-TGT resin (loading = 0.21 $mmolg^{-1}$). The resin was transferred to an automated peptide synthesiser (ABI 433A) reaction vessel for peptide chain elongation employing the Fastmoc^{TM} protocol: 10 equivalents of standard Fmoc-Xaa-OH, HBTU/HOBt as coupling reagents and DIPEA as base. After chain assembly the resin was treated with trifluoroacetic acid: ethanedithiol: water (95: 2.5: 2.5 = 5.0 mL) for 4 h. Next, the resin was filtered off and the filtrate was added to cold diethyl ether (20.0 mL), which induced precipitation, and this was centrifuged at 3000 rpm, 4 °C for 15 min. The ether layer was then decanted and the precipitated peptide was washed once more (gentle vortexing) with cold diethyl ether (20.0 mL). The white precipitate was then dissolved in water and purified by semi-preparative reverse phase (RP)-HPLC (gradient: 5$\rightarrow$50 % acetonitrile/ 45 min). Fractions containing product were identified by LC-MS, pooled and lyophilised to afford pure peptide products in multi- milligram quantities as fluffy white solids.

^{1}H NMR spectrum of isolated model peptide hydrazide **(2)**

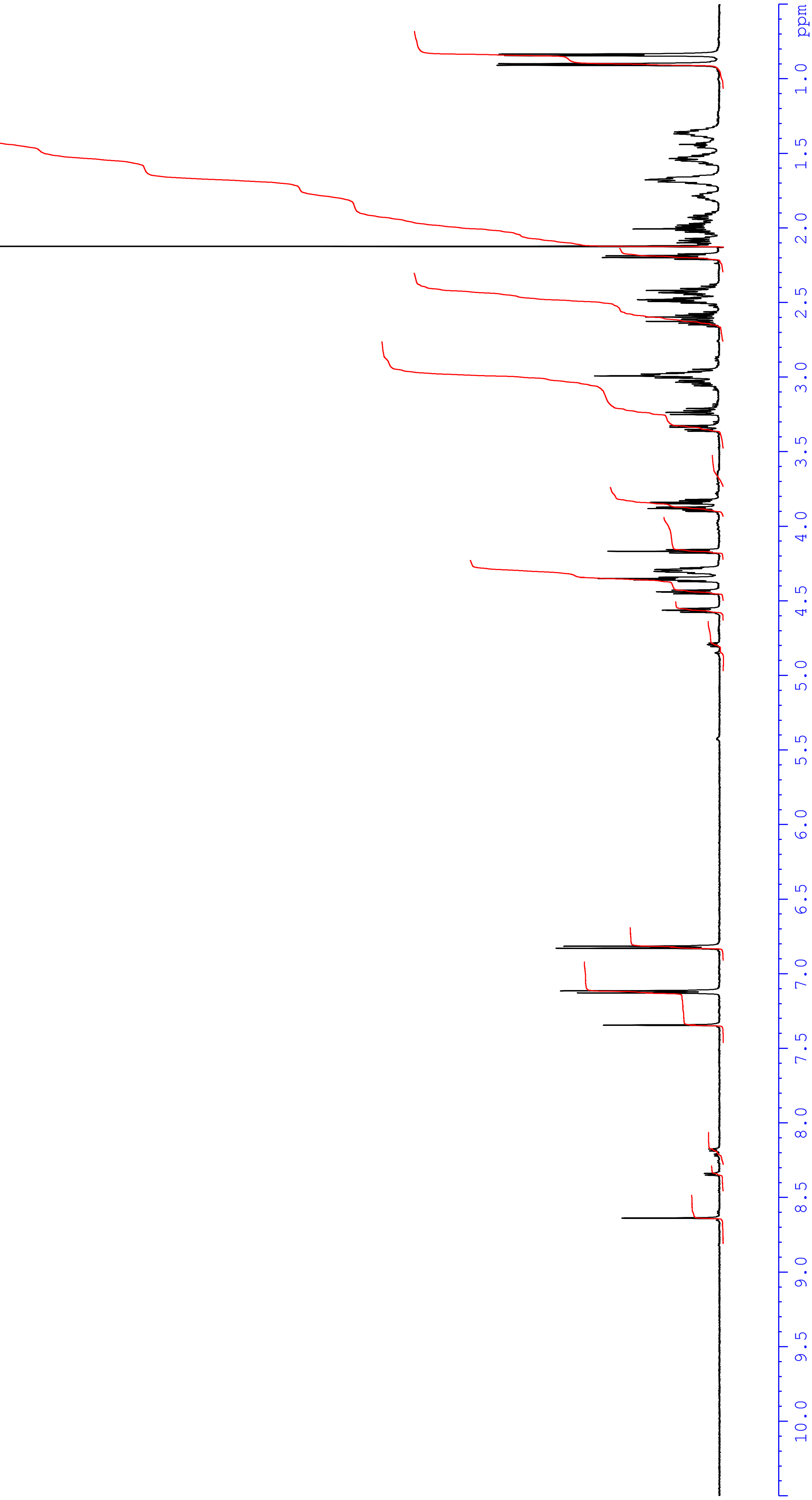

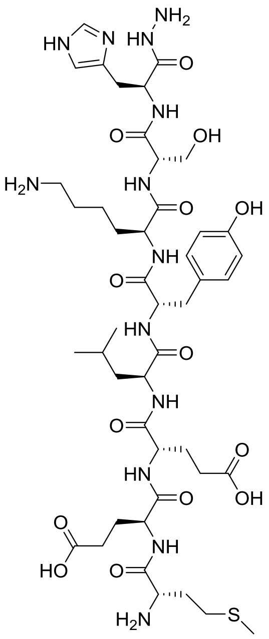

2

Model peptide ^{13}C-NMR studies

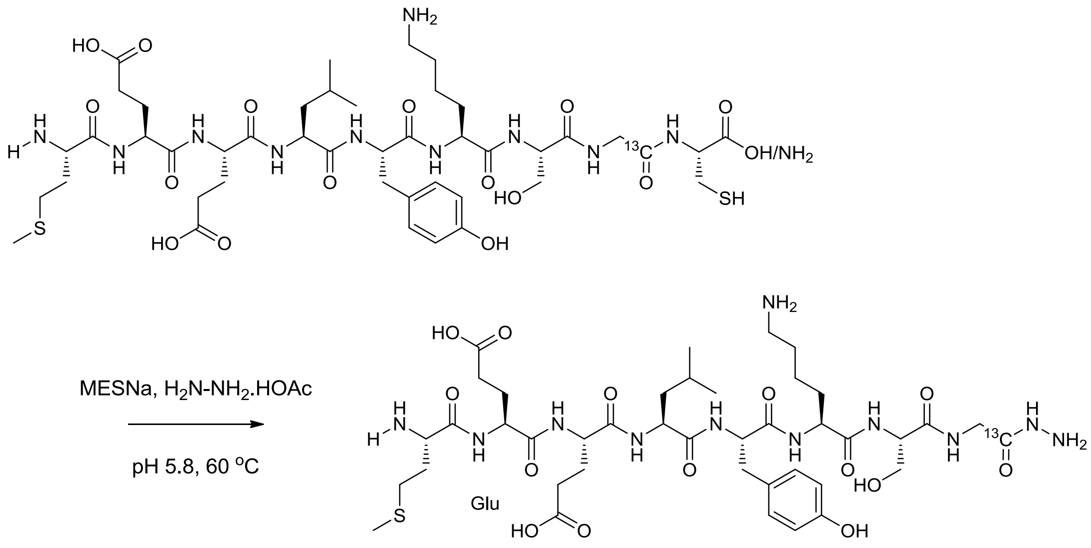

Model peptide was dissolved at 1 mg/mL concentration in 0.1 M sodium phosphate buffer (pH 5.8), MESNa (10% v/w) and hydrazine acetate (5% w/v) in D_2O. This reaction was shaken on an Eppendorf thermomixer at 600 rpm at 40- 60 °C. In order to follow hydrazide formation, 0.5 mL aliquots were removed at 0 h, 6 h, 24 h and 48 h for ^{13}C NMR analysis. Hydrazide was formed in both cases, complete in 24 h for model-OH and in 48 h for model-NH_2. ^{13}C NMR: (D_2O, 150 MHz) δ_C/ppm: 172.6 (-*C*ONH-), calculated mass: 971.1 Da, observed mass (ESI-MS) $[MH]^+$ 971.4 Da. **Note:** At lower concentrations of hydrazinium acetate (1% w/v) both hydrazide and thioester could be observed by ^{13}C NMR. However, reducing the hydrazinium acetate concentration to 2.5% w/v had little effect on the outcome at 50 ^{o}C.

<u>Synthesis of internal Gly-Cys ^{13}C-labeled model peptide:</u>

Peptide H-MEELG(^{13}C-1)CYKS-OH was manually prepared using preloaded Fmoc-Ser(Trt)-NovaSyn TGT resin , loading = 0.24 mmol/g (208 mg, 0.05 mmol). The resin was placed in a sintered filter tube and treated with 20% piperidine (v/v) in DMF (2 mL, 15 min), filtered, and exhaustively washed with DMF and CH_2Cl_2. Fmoc-Lys(Boc)-OH (5 eq, 117 mg, 0.25 mmol) was then coupled in a reaction mixture comprising O-benzotriazole-N,N,N',N'-tetramethyl-uronium-hexafluoro-phosphate (HBTU)/hydroxybenzotriazole (HOBt) solution (0.45 M, 0.55 mL) and N,N-diisopropylethylamine (DIPEA) (75 µL) in dry DMF (0.55 mL) on a horizontal shaker (400 rpm) at room temperature for 4 h. The resin was filtered-off, washed exhaustively with DMF and CH_2Cl_2, and the Fmoc deprotection/coupling process was repeated for each amino acid in the sequence. Following the final Fmoc deprotection step, the peptide was cleaved from the solid support as described above and purified by rp-HPLC to yield the peptide as a white fluffy solid (20.7 mg, 39%), ^{13}C-NMR: (100 MHz; D_2O) δ_C/ppm: 172.0 (-*C*ONH-), t_R 24.5 min, calculated mass (*m/z*): 1059.2, observed mass (ESI-MS) (*m/z*) $[MH]^+$ 1060.5.

Figure S1: ^{13}C NMR analysis of thioester formation from ^{13}C-Gly thioester precursor

H-MEELYKS**G**C-OH/NH_2

Hydrazinolysis of ^{13}C-1 Gly-Cys carboxylic acid terminated model peptide (sequence MEELYKS**G**C)

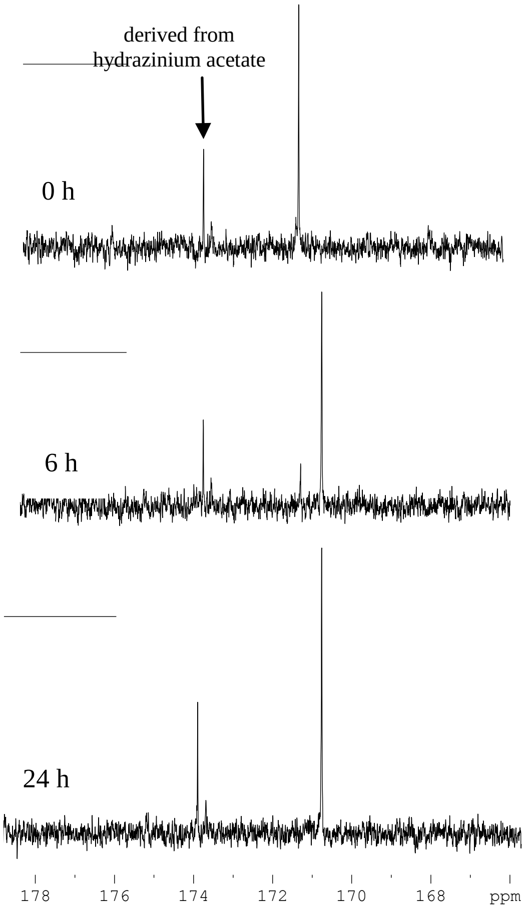

Hydrazinolysis of ^{13}C-1 Gly-Cys carboxamide terminated model peptide (sequence MEELYKS**G**C)

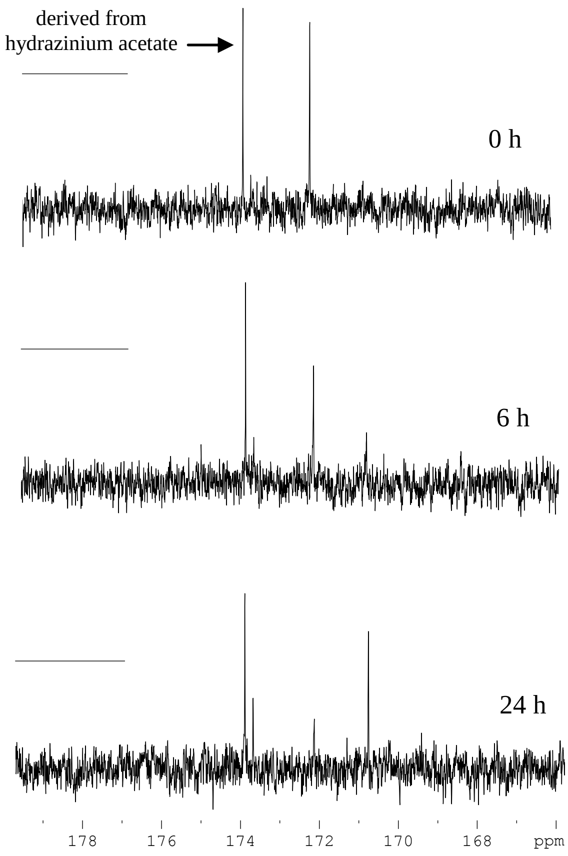

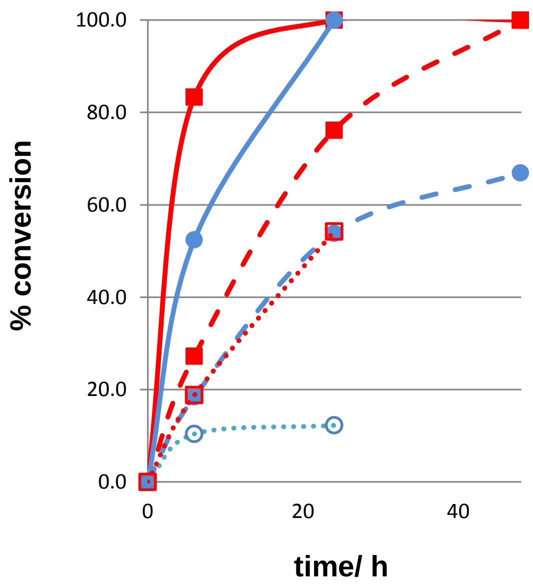

^{13}C NMR comparison of hydrazinolysis (red) and thioester formation (blue) in the same model peptide at 60 °C.

Solid lines correspond to hydrazinolysis/thioester formation of **carboxyl terminated** peptide and the dashed lines correspond to hydrazinolysis/thioester of **carboxamide terminated** peptide.

Dotted lined correspond to hydrazinolysis/ thioester formation of an **internally situated** model system: sequence: H-MEEL**GC**YKS-OH (monitored for 24 h only)

Figure S2: Conversion of **2** to the corresponding MESNa thioester:

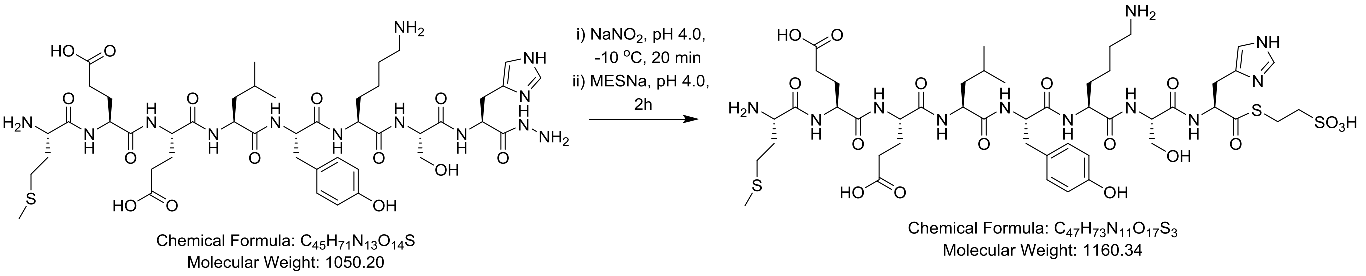

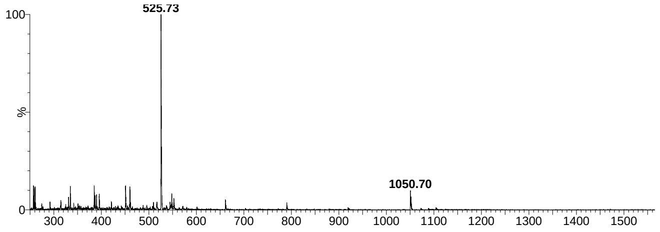

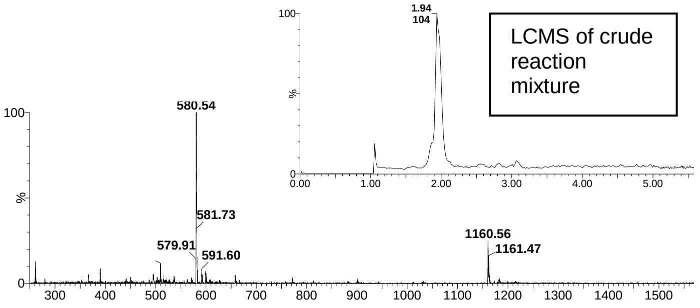

Hepcidin Hydrazinolysis Reactions

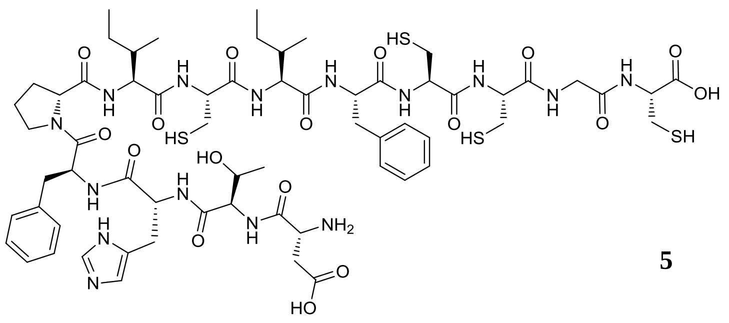

5

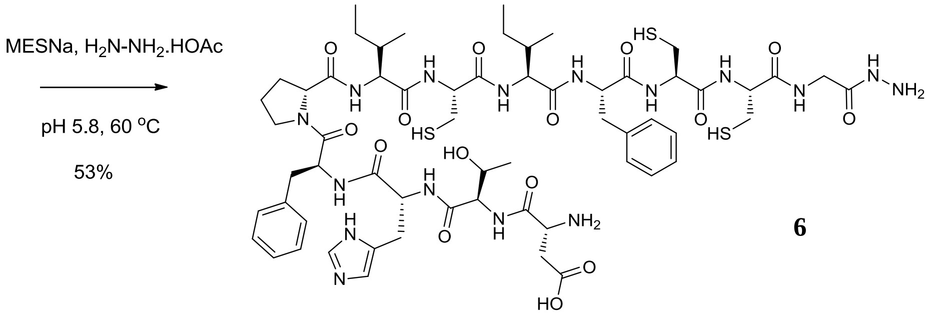

6

Peptide **5** (30 mg, 17.1 µmol), was dissolved at 1 mg/mL concentration in 6.0 M guanidine hydrochloride (final volume 30 mL) with 0.1 M sodium phosphate buffer pH 5.8, MESNa (10% w/v) and hydrazine acetate (5% w/v). This reaction was shaken on an Eppendorf thermomixer at 600 rpm at 60 °C for 48 h. The reaction was followed by LC-MS and peptide hydrazide was isolated by HPLC and lyophilised to yield the peptide hydrazide **6** as a white fluffy solid (15.9 mg, 53%), t_R 28.8 min, calculated mass: 1582.9 Da, observed mass (ESI-MS) $[MH]^+$ 1582.8 Da.

Ligation of Hepc1-12NHNH$_2$ and Hepc13-25

7

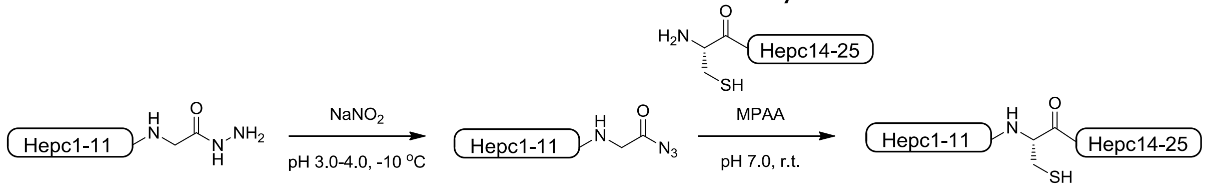

6

8

6 (3.2 mg, 2.02 µmol) was dissolved in ligation buffer comprised of 0.2 M sodium phosphate buffer pH 3.0-4.0 in 6.0 M guanidine hydrochloride (0.7 mL). This solution was cooled to -10 °C and the pH was checked. Oxidative solution containing sodium nitrite (70 µL of 0.2 M stock solution) was added dropwise to the reaction mixture. The reaction was then stirred at -10 °C for 20 min. After this, neutral ligation buffer was added, comprising 0.2 M sodium phosphate buffer pH 7.0, **7** (4.6 mg, 2.62 µmol) and 0.2 M mercaptophenyl acetic acid (MPAA) in 6.0 M

guanidine hydrochloride (0.7 mL). The reaction was left to stir at room temperature overnight and monitored by LC-MS. Once the reaction appeared complete, it was reduced using TCEP (30 mM) for 1 h at room temperature before being purified by rp-HPLC to yield the ligated peptide **8** as a white fluffy solid, t_R 27.5 min, calculated mass, 3295.0 Da; Observed mass (ESI-MS) 1099.5 Da $[MH]^{3+}$ which deconvolutes to 3295.5 Da.

Removal of Protecting groups and folding of hepcidin:

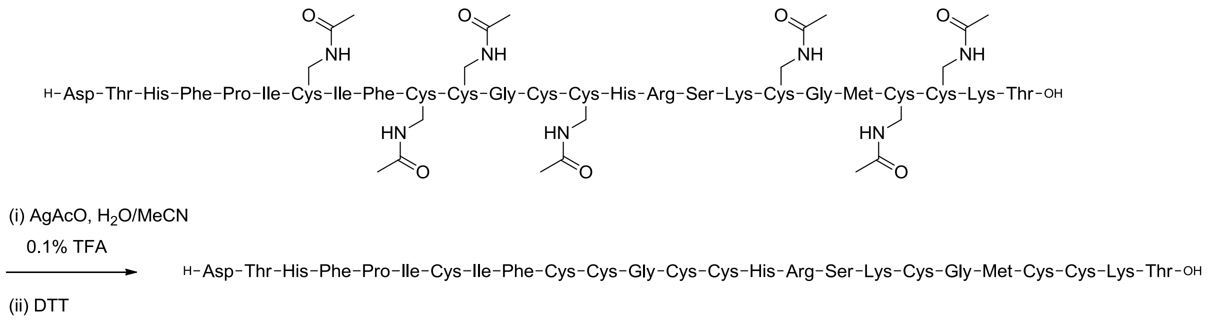

8 (7 mg, 2.1 µmol) was dissolved at 1 mg/mL in a solution of 1:1 acetonitrile/water (2.1 mL) containing 0.1% v/v TFA (2.1 µL). To this was added silver acetate (10 eq per protected cysteine, 23 mg, 140 µmol, 70 eq), and the reaction was shaken at room temperature for 3 h. DTT (100 mg) was then added and the reaction was left to shake at room temperature overnight. Deprotection was confirmed by LC-MS (hexathiol was observed by LC-MS prior to rp-HPLC, calculated mass, 2797.4 Da; observed mass (ESI-MS) 933.0 Da $[MH]^{3+}$ which deconvolutes to 2796 Da). The resultant mixture was centrifuged and the supernatant purified by rp-HPLC.

Fractions containing the deprotected peptide were collected from the HPLC and diluted using 40% acetonitrile/water to give an estimated concentration of 0.16 mg/mL of peptide. The pH was adjusted to pH 7.5 using 30% ammonium hydroxide solution and 3 equivalents each of oxidized and reduced glutathione were added. The reaction was shaken gently overnight at room temperature, then neat trifluoroacetic acid was added to lower the pH to pH 2. The solution was lyophilized, redissolved in 30% acetonitrile/water and purified by rp-HPLC.

Expression of ubiquitin:

The WT ubiquitin clone was supplied by Dr L. Cabrita and Dr J. Christodoulou in a pET2b(+) plasmid. The ubiquitin G76C mutant was cloned from the WT ubiquitin pET2b(+) plasmid into a pNIC28-Bsa4 vector with TATCCACCTTTACTGTCAACAACCACGTAGACGTAAGAC as the forward and TATCCACCTTTACTGTTAACAACCACGTAGACGCAAGAC reverse primers respectively. DNA sequencing confirmed the identity of the construct and the vector was heat-shock transformed into *E. coli* BL21 (DE3) cells for expression.

Cells were grown at 37°C in 1 L of Luria-Bertani media supplemented with Kanamycin (50 μgL^{-1}). Gene expression was induced, once an OD_{600} of 0.6 was reached, by addition of 1 mM isopropyl-β-D-thiogalactopyranoside. The culture was maintained for a further 16 hours at 22°C. Cells were harvested by centrifugation at 4,000 g for 30 min at 4°C. The cells were resuspended into a lysis buffer (20 mL, 50 mM sodium phosphate buffer, pH 7, 250 mM NaCl, 25 mM imidazole, 1 mM TCEP) containing DNAse I (0.2 mg) and two tablets of a cocktail of EDTA-free protease inhibitors (Roche). Cell lysis was achieved through sonication (6 x 30s burst with 1 min cooling intervals). The resultant lysate was centrifuged at 35,000 g for 30 min at 4°C and the supernatant loaded onto a pre-equilibrated HisTrap HP 5 mL column (GE Healthcare) pre-charged with Ni^{2+}. A linear gradient of imidazole, 25 mM to 1 M was applied to elute ubiquitin. Samples were further purified using a Superdex 75 16/60 size exclusion column (GE Healthcare) equilibrated with 50 mM sodium phosphate buffer, pH 7, 250 mM NaCl, 1 mM TCEP.

The protein eluted with one peak of a mass of approximately 12 kDa corresponding to a monomer. Fractions containing the protein were pooled, exchanged into a pH 5.8 buffer (100 mM sodium phosphate buffer pH 5.8, 100 mM NaCl), using Amicon Ultra devices (Millipore) and concentrated to 1 mg mL^{-1}. The purity and identity of the protein was confirmed by mass spectrometry, with a yield of approximately 6 mg L^{-1} of cell culture.

Hydrazinolysis of recombinant ubiquitin sample

Purified ubiquitin was obtained at 1-2 mg/mL concentration, in 0.1 M NaCl, 0.1 M Na Phosphate buffer solution (pH 5.8, 0.9 mL) by use of a cerntifugal filter equipped with a 3KDa molecular weight cut off. 2-Mercaptoethanesulfonate (100 mg) was added followed by *tris*(2-carboxyethyl)phosphine (approximately 5 mg) and 100 μL of a 50% w/v aqueous hydrazinium acetate stock solution. The reaction mixture was shaken (500 rpm) at 45 °C for 48 h in an Eppendorf thermomixer. LC-MS analysis of 4 µL aliquots of reaction mixture was performed at t=0 h, t= 24 h and t= 48h and indicated that the reaction had neared completion within 48 h. The protein was then buffer exchanged, by use of a centrifugal filter equipped with a 3KDa molecular weight cut off, into "ligation buffer" (0.1 M NaCl, 0.2 M Na phosphate buffer solution; pH 4) by repeated dilution/concentration and ultimately obtained in the original reaction volume of ligation buffer (0.9 mL)

Ubiquitin C-terminal labelling:

i) with Biotin Tag (9). Ubiquitin C-terminal hydrazide (obtained as above)**,** dissolved at 1-2 mg/mL concentration, in ligation buffer (pH 4, 0.25 mL) was cooled to 0 ^{o}C with gentle magnetic stirring. Sodium nitrite 4 µL of a 0.2 M stock solution in water was added and stirring was continued at 0 ^{o}C for 20 minutes. During this time a solution comprising 0.2 M Na Phosphate buffer; pH 7, 0.1 M NaCl, 0.2 M mercaptophenylacetic acid (MPAA), and containing the biotin-tag (**9**) dissolved to a final concentration of 1 mg/mL was prepared. The MPAA/Biotin solution (0.25 mL) was added to the diazotization reaction mixture and stirring was continued at room temperature for 1 h, after which time LC-MS analysis indicated that the reaction was complete. The protein was next buffer exchanged by use of a centrifugal filter equipped with a 3KDa molecular weight cut off, into 0.1 M NaCl, 0.1 M Na phosphate buffer solution; pH 7, containing 1 mM DTT by repeated dilution/concentration and was ultimately obtained in the original reaction volume (0.25 mL).

LC-MS after 1h reaction

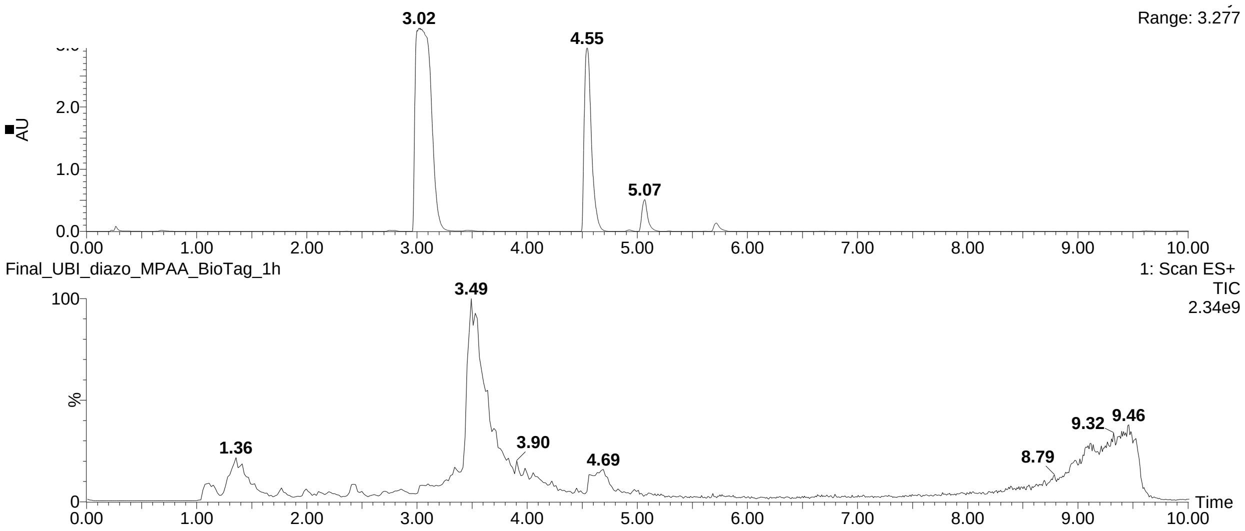

Major species at 3.5 min:

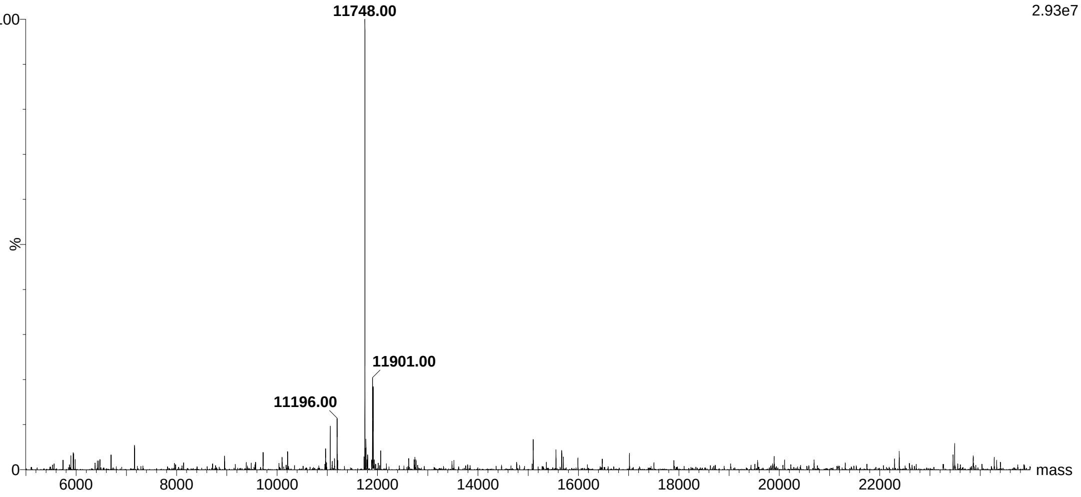

ii) with cysteamine (10). Ubiquitin C-terminal hydrazide (obtained as above), dissolved at 1-2 mg/mL concentration, in ligation buffer (pH 4, 0.25 mL) was cooled to 0 ^{o}C with gentle magnetic stirring. Sodium nitrite 4 uL of a 0.2 M stock solution in water was added and stirring was continued at 0 ^{o}C for 20 minutes. During this time a solution comprising 0.2 M cysteamine, 0.2 M Na Phosphate buffer; pH 7, 0.1 M NaCl was prepared. The cysteamine solution (0.25 mL) was added to the diazotization reaction mixture and stirring was continued at room temperature for 1 h after which time LC-MS analysis indicated that the reaction was complete. The protein was finally buffer exchanged by use of a centrifugal filter equipped with a 3KDa molecular weight cut off, into 0.1 M NaCl, 0.1 M Na phosphate

buffer solution; pH 7, 1 mM DTT by repeated dilution/concentration and ultimately obtained in the original reaction volume of ligation buffer (0.25 mL)

LC-MS analysis upon reaction with cysteamine for 1 h.

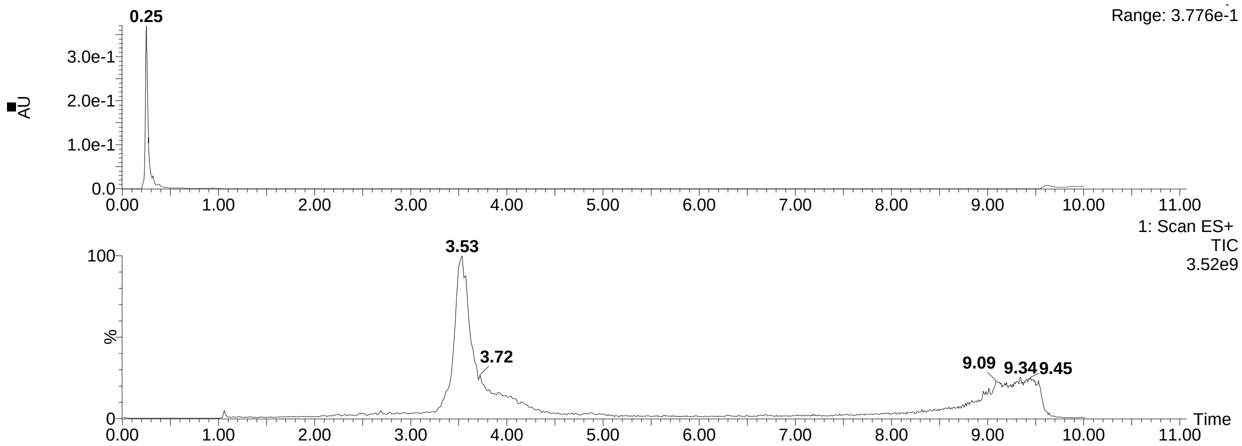

LC-MS of Major species at 3.5 min:

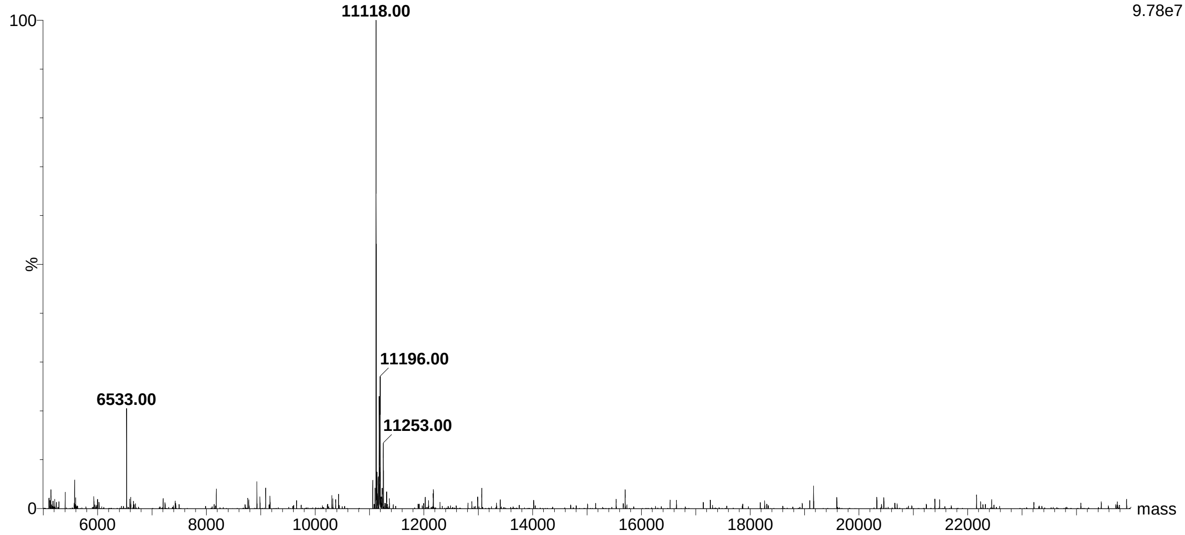

NMR analysis of ubiquitin hydrazinolysis and thioester formation/ligation. To confirm that ubiquitin remained folded, via NMR spectroscopy, 1 D ^{1}H NMR spectra were obtained. Samples were buffer-exchanged into 0.1 M sodium phosphate pH 5.8 (containing 1 mM TCEP), and supplemented with 10% D_2O. Data acquisition was carried out on a 500 MHz Bruker Avance III spectrometer. Following each procedure the backbone amide region (7-10ppm) remained largely unchanged.

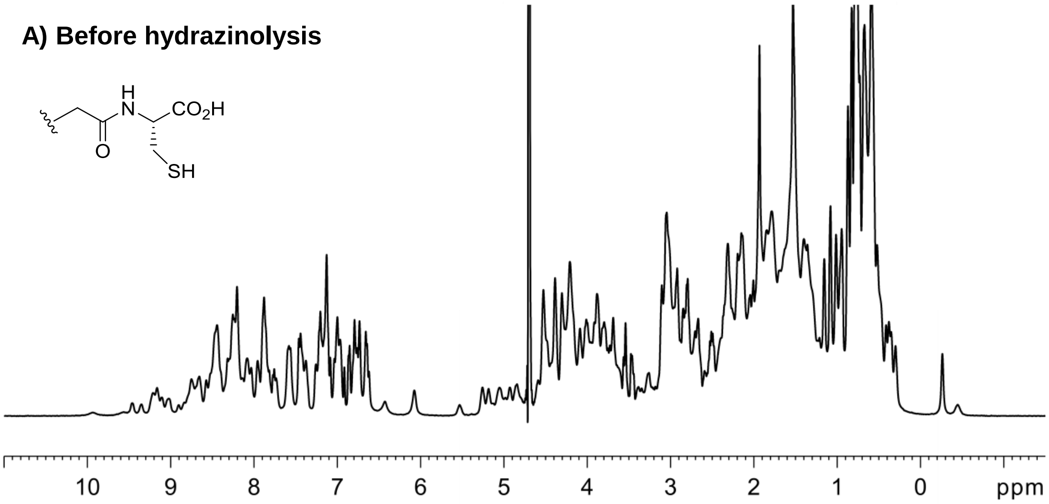

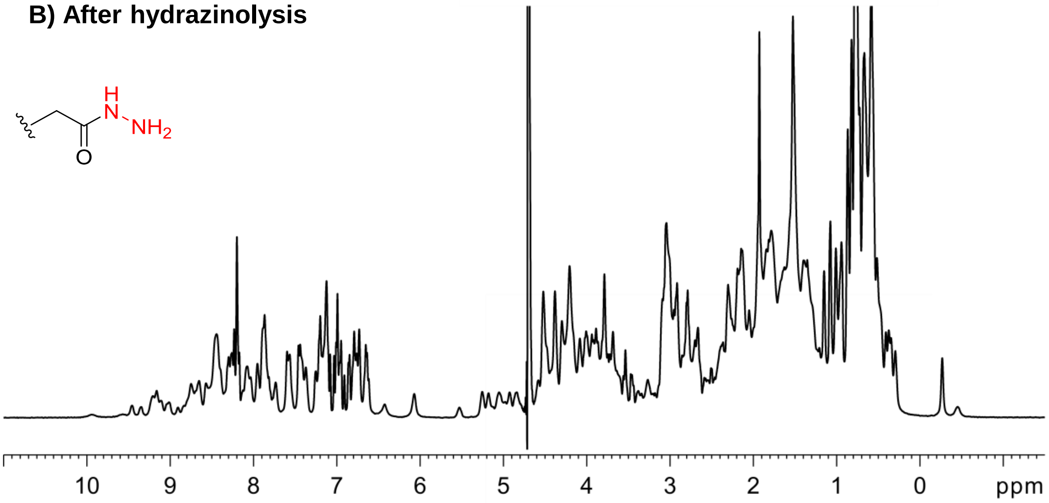

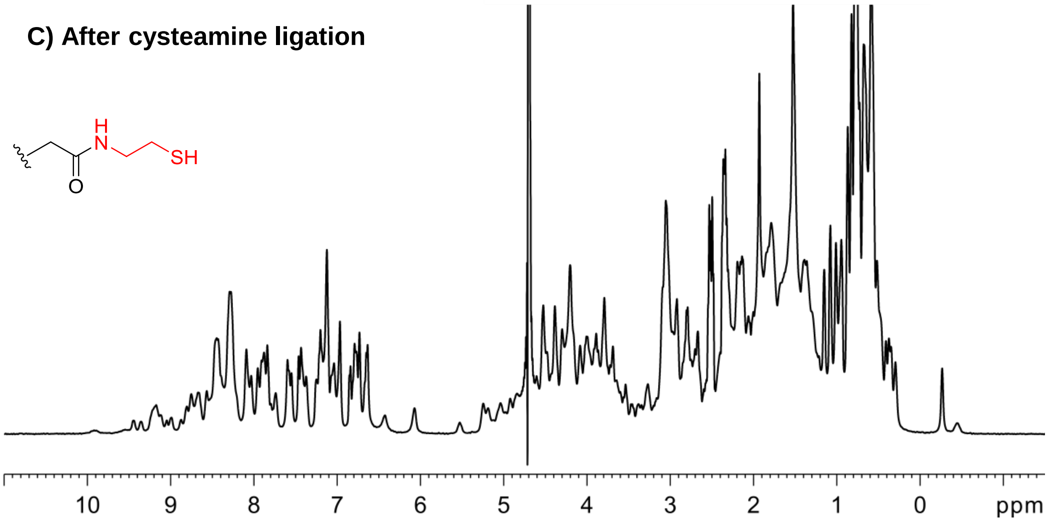

Production of EPO residues 1-160

The DNA sequence encoding EPO residues 1-161(A160G) were amplified from a template encoding full-length EPO, via PCR. Primers $^{5'}$ggtggtcatatggccccaccacgcctc (forward) and $^{5'}$ggtggtggatccttagcacccctcccctgtgtacag (reverse) were designed to respectively incorporate NdeI and BamHI restriction endonuclease sites, enabling digestion and ligation into pET-16b expression vector, using a Rapid Ligation Kit (Roche). His-tagged EPO1-161(A160G) was expressed in B834(DE3) *E. coli* cells via incubation of 500ml LB broth cultures (containing 100 µg/ml ampicillin) at 37°C until an OD_{600} of approximately 0.6, followed by addition of isopropyl-β-D-1-thiogalactoside (Sigma-Aldrich) (1 mM final concentration) and continued incubation at 37°C for a further 3 hours. Cells were harvested via centrifugation, resuspended in lysis buffer (20 mM Tris pH 7.9, 0.5 M NaCl, 5 mM imidazole) and lysed via sonication in the presence of 1 mM PMSF. Insoluble material was then pelleted via centrifugation at 13,000 RPM, resuspended in lysis buffer containing 6M guanidine hydrochloride, and unbroken cells and debris removed via centrifugation at 19,000 RPM. His-tagged EPO1-161(A160G) was purified through immobilised metal-ion affinity chromatography (IMAC) using Ni-NTA agarose (Qiagen) via gravity-flow. The eluted protein was precipitated via dialysis against 4 litres of distilled water for 18 hours at 4°C. Precipitated EPO1-161(A160G) (5 mg/ml) was resuspended in 6M guanidine hydrochloride containing 1% TCEP hydrochloride. The protein was further purified via RP-HPLC, using a Dionex Ultimate 3000 system equipped with a Phenomenex Jupiter 10µ Proteo 90A, C_{12}, 250 x 21.2 mm column, using a mobile phase of 0.1% TFA (v/v) in water /acetonitrile over a 5-60% acetonitrile gradient. Fractions contianing the protein fragment were identified by LC-MS and lyophilized to obtain the product as a fluffy white solid.

Hydrazinolysis was then carried out by dissolving the protein in 6M guanidine hydrochloride containing 1% TCEP hydrochloride. MESNa was then added to a final concentration of 10% w/v, followed by hydrazine acetate (5% w/v) and the reaction pH adjusted to pH 5 if necessary. Reactions were incubated at 45°C for 48 hours, followed by buffer-exchange into 6M guanidine using a 3000 MWCO centrifugal concentrator. Reactions were replenished with fresh reagents, and resumed for a further 48 hours.

Note 1: Alternative solubilization buffer systems to 6 M guanidine.HCl, such as 6 M urea, 1 % sodium dodecyl sulfate, n-ocyl maltoside, and 2 % N-lauroyl sarcosine were also examined for the solubilisation and hydrazinolysis of EPO without success.

Note 2: For synthesis purposes hydrazinium acetate can be replaced with hydrazine hydrate or hydrazine dihydrochloride, but the pH of stock solutions of these reagents must first be adjusted (with

HCl or NaOH respectively) to within the range of the reaction (pH 5- pH 6) prior to addition to the reaction mixture.
